# What do young adult Italians know about STIs? Evidence from a national survey

**DOI:** 10.3389/fpubh.2026.1947644

**Published:** 2026-10-13

**Authors:** Silvia Cocchio, Andrea Cozza, Matilde Obici, Elisabetta Conte, Claudia Cozzolino Cangiano, Nicoletta Parise, Barbara Morsello, Stefano Crabu, Cosimo Marco Scarcelli, Patrizia Furlan, Federico Neresini, Vincenzo Baldo

**Affiliations:** 1 Department of Cardiac, Thoracic, Vascular Sciences, and Public Health, University of Padua, Padua, Italy; 2 Preventive Medicine and Risk Assessment Unit, Azienda Ospedale Università Padova, Padua, Italy; 3 Department of Philosophy, Sociology, Education and Applied Psychology, University of Padua, Padua, Italy

**Keywords:** condom, knowledge, prevention, protective behaviors, sexually transmitted infections

## Abstract

**Background:**

Sexually transmitted infections (STIs) represent one of the major challenges for global public health. Across Europe, surveillance data indicate a concerning increase in cases of gonorrhea, syphilis, and chlamydia; in Italy, sentinel surveillance data similarly show an increase in overall STIs reports. Despite this alarming trend, the literature highlights poor awareness of STIs among the Italian population, particularly among young adults. This study aims to investigate the level of knowledge about STIs and protective behaviors among young Italians.

**Methods:**

A questionnaire was administered to young Italians aged 16–30 years, residing in four Italian geographical areas (northwest, northeast, center, and south), in December 2023. The questionnaire included 14 items on STIs knowledge with response options “True,” “False,” or “I don't know.” Knowledge was measured using a weighted continuous score and classified into four categories (low, moderate-lower uncertainty recognition, moderate-higher uncertainty recognition, high). Sexual behaviors and condom use were also analyzed.

**Results:**

A total of 2,576 young people responded to the questionnaire. Only 12.5% of participants achieved a high level of STIs knowledge, with an average weighted correct answer rate of 37.07%. Knowledge was significantly lower among males, younger individuals, and those with lower educational levels. Among the 1,653 young people who reported being sexually active in the past year, 33.8% reported always using condoms. The main reasons for non-use were: trust in their partner (40.5%), embarrassment (30%), and the fact that their partner disliked it (30.8%). Among those with high STIs knowledge, condom use remained low due to emotional-relational reasons.

**Discussion:**

The study highlights a low level of STIs knowledge among young Italians and insufficient condom use. The results suggest that increasing knowledge alone is not sufficient to modify behaviors: it is also necessary to address psychological, cultural, and relational barriers. Multidisciplinary interventions in schools, targeted information campaigns, and sexual and emotional education are recommended.

## 1 Introduction

The World Health Organization (WHO) estimates that HIV, viral hepatitis, and sexually transmitted infections (STIs) currently represent one of the major public health problems, contributing, globally, to a high disease burden (1, 2). Over 30 pathogens are responsible for STIs (1, 2). The main bacterial STIs include chlamydia, gonorrhea, and syphilis. The major viral STIs include HIV, HSV, HBV, HPV, and HTLV-1 (2, 3). Protozoan and parasitic infections such as trichomoniasis are also included (2). Historically, the main sexually transmitted diseases were syphilis, gonorrhea, lymphogranuloma venereum, and chancroid. Today, these have largely been replaced by second-generation STIs caused by viruses (HIV, HSV, HPV, hepatitis viruses), chlamydia, trichomonas, and the resurgence of syphilis and gonorrhea (4).

In 2020, the WHO estimated 374 million new STIs globally among individuals aged 15–49; of these, one in four was attributable to a treatable bacterial/protozoal infection among *Chlamydia trachomatis, Neisseria gonorrhoeae, Treponema pallidum* and *Trichomonas vaginalis* (5). At the end of 2022, it was estimated that globally there were approximately 39 million people living with HIV, with 1.3 million new cases recorded. Regarding hepatitis, in 2022 it was estimated that a total of 304 million people were affected by hepatitis B and C (254 million and 50 million, respectively), with 2.2 million new cases occurring during the year. Globally, in 2020, 3.8 billion people under the age of 50 were estimated to have an HSV-1 infection (oral or genital); for genital herpes caused by HSV-2, approximately 520 million individuals aged 15–49 years were estimated to be infected in 2020 (6). Currently, more than 500 million people aged 15 to 49 worldwide have a genital herpes simplex infection (7). In 2007, it was estimated that 291 million women carried HPV DNA.

At the European level, in 2019, 117,881 cases of gonorrhea, 434,184 cases of chlamydia, and 35,039 cases of syphilis were reported (8–10). Furthermore, in 2022, an increase in new cases was recorded: gonorrhea increased by 48%, syphilis by 34%, and chlamydia by 16% compared to 2021 (11).

In Italy, a similar upward trend has been observed in overall STIs reports, based on data mainly derived from two active sentinel surveillance systems: the Clinical Surveillance System and the Microbiological Surveillance System. The Clinical Surveillance System, active since 1991, integrates data from a network of 12 public centers specializing in the care of individuals with STIs. The Microbiological Surveillance System, active since 2009, collaborates with a network of 12 clinical microbiology laboratories, which report new cases of specific STIs such as *Chlamydia trachomatis, Trichomonas vaginalis*, and *Neisseria gonorrhoeae* (12). Both surveillance systems are coordinated by the AIDS Operational Center of the *Istituto Superiore di Sanità* (e.g., National Institute of Health). From 1991 to 2021, the surveillance system reported a total of 151,384 new cases of STIs. The number of STIs cases remained stable until 2004, with an average of 3,994 reported STIs cases per year; from 2005 to 2016, reports increased by 37.4% compared to the 1991–2004 period; in 2021, reports (5,761 STIs cases) increased by 17.6% compared to 2020 (4,748 STIs cases) (12). This increase in STIs cases indicates a trend opposite to respiratory diseases, which have numerically decreased (13).

STIs have a significant impact on human reproductive health and represent one of the most significant challenges for global health. Beyond their physical consequences, STIs may also affect psychological wellbeing, quality of life, and sexual functioning, with diagnosed individuals potentially experiencing stigma, anxiety, depressive symptoms, and relational difficulties (14–16).

Regarding knowledge about STIs, literature data show that the Italian population generally has a low overall awareness of STIs, with differences related to sex assigned at birth (higher awareness among women than men), type of STIs, and regional distribution (north-south gradient) (17–19). Specifically, for STIs types, there is generally greater awareness of HIV, likely due to educational and preventive campaigns over the past 30 years. However, the literature still highlights inadequate awareness of the problem (17).

Regarding preventive strategies, a recent WHO globally report reveals a worrying decline in condom use among adolescents, with a consequent increase in the risk of sexually transmitted infections (20).

The literature analysis suggests that Italy tends to have limited awareness of the risks associated with sexual activity and common sexually transmitted infections and of prevention strategies (17, 19, 21–23). Despite the public health relevance of STIs among young people, recent evidence jointly examining STIs knowledge, its associated factors, condom-use frequency, and the reported reasons for condom non-use across different geographical areas of Italy remains limited.

### 1.1 Aim of study

The aim of the study is to assess the level of knowledge about STIs in a sample of young Italians and investigate which variables are associated with it. Additionally, condom use as a preventive measure and the profile of those who do not use it were evaluated.

## 2 Materials and methods

### 2.1 Study design

A specifically tailored questionnaire was created to investigate young people's perspectives on issues related to sexuality. It was administered in December 2023 to Italian individuals aged 16–30 living in four geographical regions with comparable prevention strategies and vaccination policies (northwest, northeast, central, and southern Italy) (24). The questionnaire included 24 items divided into five sections. The present study focused on the analysis of demographic characteristics, general knowledge of sexually transmitted infections, and risk or protective behaviors, such as condom use. All data were collected anonymously.

The STIs knowledge section comprised 14 items with response options “True,” “False”, or “I don't know”. Knowledge was measured using (i) a continuous score, defined as the sum of correct answers weighted by an item difficulty index (the proportion of incorrect or “I don't know” responses in the full sample), and (ii) a categorical measure that accounted for both correct and ‘I don't know' responses. We defined four categories: “Low” (correct answers ≤ 3/14), “Moderate-lower uncertainty recognition” (correct answers between 4–9 and “I don't know” ≤ incorrect answers), “Moderate-higher uncertainty recognition” (correct answers between 4–9 and “I don't know” > incorrect answers), and “High” (correct answers ≥10/14).

The “Education Level” variable was categorized as “Years of study” (< 13 years; 13 years; >13 years). Given the overrepresentation of participants with higher educational attainment, we used post-stratification weighting to calibrate the sample to the resident-population distribution by age group, education level, and gender, as reported by the Italian National Institute of Statistics (ISTAT; *Istituto Nazionale di Statistica*). This weighting factor was applied in all analyses.

Non-binary participants were not included in the analyses because the ISTAT population data used for post-stratification did not provide a corresponding category, precluding the calculation of an appropriate weighting factor. Including these participants without a valid population benchmark would have been inconsistent with the weighting procedure applied to the rest of the sample.

Data obtained from the questionnaire were summarized as means ± standard deviations (SD) for continuous variables and as percentages for categorical variables. Comparisons of continuous data were performed using the Student's *t*-test or analysis of variance (ANOVA), whereas categorical data were evaluated using the Chi-square test.

For the main covariates of interest, the average number of weighted correct answers (on a scale from 0 to 14 and as a percentage from 0 to 100) was calculated. To assess which variables were significantly correlated with the level of knowledge, a multiple linear regression was performed, with the weighted number of correct answers as the dependent variable and age, sex assigned at birth, sexual orientation, gender identity, educational level, type of employment, citizenship, economic condition, STIs diagnosis (yes/no), geographical area, and participation in sexual education courses as independent variables. The correlation was estimated using the β coefficient (95%CI) with a 95% confidence interval.

Among respondents who reported having had sexual intercourse in the previous year, condom-use frequency was classified into the following categories: “Always,” “Often,” “Occasionally/sometimes,” and “Never”; non-responses were also recorded. The reasons for not using condoms were analyzed by stratifying responses according to sex assigned at birth, frequency of condom use (“Never” or “Occasionally/sometimes”), and overall level of STIs knowledge (“Low,” “Moderate—lower uncertainty recognition,” “Moderate—higher uncertainty recognition,” and “High”). To identify factors associated with occasional or no condom use, a logistic regression analysis was performed comparing participants who reported “Always” using condoms with those who reported using them “Occasionally/sometimes” or “Never”. Participants who responded “Often” were excluded from the analysis because this response represented an intermediate and heterogeneous category that could not be unambiguously classified as either consistent condom use or occasional or no condom use. The analysis was therefore designed to clearly distinguish consistent condom use from occasional or no condom use. Participants who did not answer the question on condom-use frequency were also excluded from the regression analysis. The model was adjusted for the following variables: age, sex assigned at birth, gender identity, sexual orientation, geographical area, citizenship, employment status, economic condition, level of STIs knowledge, and sexual behaviors, including oral and anal sex.

Adjusted ORs (adjOR) with their respective 95% confidence intervals were estimated.

A *p*-value < 0.05 was considered statistically significant. Analyses were conducted using the Statistical Package for the Social Sciences (SPSS 28.0; SPSS Inc., Chicago, USA).

### 2.2 Ethics committee

This study was conducted in accordance with the Declaration of Helsinki. Data collection was conducted in December 2023 by an external professional survey company, formally designated as Data Processor pursuant to Article 28 of the GDPR (General Data Protection Regulation). Questionnaire responses were anonymized at the source before being made available to the research group. The research team was not involved in participant recruitment or in the direct administration of the questionnaire. The authors' research activity consisted exclusively of the subsequent use and analysis of the anonymized dataset. The research project concerning the use and analysis of these data was subsequently submitted to the Research Ethics Committee of the FISPPA Department, University of Padua, which issued a favorable opinion on 3 April 2024 (Reference Number: Prot. N. 0001419 – 03 April 2024). The anonymized dataset was made available to the research team and analyzed for research purposes following receipt of this ethical approval.

## 3 Results

### 3.1 Participant characteristics

The total study sample consisted of 2,576 subjects. The average age was 23.2 ± 3.9 years; specifically, 22.1% were aged between 16 and 20 years, and 77.9% were aged between 20 and 30 years. Of the total sample, 51.7% were male (*n* = 1,333), 96.1% (*n* = 2,474) identified with their biological sex (cisgender), 81.5% (*n* = 2,100) reported a heterosexual orientation, and 94.4% (*n* = 2,433) held Italian citizenship. Overall, 9.2% (*n* = 238) reported having received an STIs diagnosis, 88.3% (*n* = 2,274) reported never having received one, while 2.5% (*n* = 64) did not respond (nr). The average age was significantly higher among those who reported an STIs diagnosis (24.2 ± 4.0 years) compared to those who did not (23.1 ± 3.9 years) (*p* < 0.001). The frequency of self-reported STIs diagnoses was higher among transgender individuals (25.6% vs. 8.6%), non-heterosexuals (15.9% vs. 7.7%), and foreign citizens (15.2% vs. 8.9%).

Regarding the level of education, 34.3% of respondents (*n* = 882) had less than 13 years of schooling, 49.0% (*n* = 1,262) had 13 years, and 16.7% (*n* = 431) had more than 13 years. A total of 38.2% (*n* = 985) were employed, and 43.9% reported difficulty making ends meet at the end of the month (“some difficulty” 34.3%; “major difficulty” 9.6%). Bivariate analysis showed a significantly higher frequency of STIs self-diagnosis among those with lower education levels (12.8% vs. 7.3% and 7.7%), among employed individuals compared to those not in the workforce (10.6% vs. 7.7%), and among those in more favorable economic conditions compared to those with significant economic difficulties (18.2% vs. 10.7%). Of the respondents, 39.4% were from the North, 40.4% from the Center, and 20.2% from the South, with no differences between those who reported an STIs diagnosis and those who did not.

Regarding sexual behaviors, 70.7% of the sample reported engaging in oral sex, and 38.5% reported engaging in anal sex, with a higher frequency of STIs self-diagnosis (11.3% vs. 4.4% and 15.2% vs. 5.5%, respectively). In the past year, 1,653 subjects (64.2%) reported having had sex, of whom 33.8% (*n* = 559) reported always using condoms, while 63.5% used them irregularly (often: 16.9%; occasionally/sometimes/never: 46.6%).

A total of 50.1% of the sample reported having attended sex education sessions, specifically 15.8% at school and 34.3% in non-school settings (municipalities, counseling centers, public institutions, associations, political organizations, and others) (Table 1).

**Table 1:** Sample description.

	Total		STIs diagnosis						*p*-value
			Yes		No		nr			
	*n*	(%)	*n*	(%)	*n*	(%)	*n*	(%)	
**Total**	2,576	**(100.0)**	**238**	**(9.2)**	2,274	**(88.3)**	**64**	**(2.5)**	**–**
Sex assigned at birth									
*Males*	1,333	(51.7)	125	(9.4)	1,173	(88.0)	34	(2.6)	ns
*Females*	1,244	(48.3)	113	(9.1)	1,101	(88.5)	30	(2.4)	
Gender identity									
*Cisgender*	2,474	(96.1)	212	(8.6)_a_	2,209	(89.3)_a_	53	(2.2)_a_	**< 0.001**
*Transgender*	102	(3.9)	26	(25.6)_b_	65	(64.2)_b_	10	(10.2)_b_	
Sexual orientation									
*Heterosexual*	2,100	(81.5)	163	(7.7)_a_	1,899	(90.4)_a_	38	(1.8)_a_	**< 0.001**
*not Heterosexual*	476	(18.5)	76	(15.9)_b_	375	(78.7)_b_	25	(5.4)_b_	
**Age (average** **±SD)**	23.2 ± 3.9		24.2 ± 4.0		23.1 ± 3.9		22.5 ± 4.0		**< 0.001**
Age group									
*16–19 years*	570	(22.1)	42	(7.4)_a_	507	(89.9)_a_	21	(3.6)_a_	**0.029**
*20–30 years*	2,006	(77.9)	196	(9.8)_a_	1,767	(88.1)_b_	43	(2.1)_b_	
Citizenship									
*Italian*	2,433	(94.4)	217	(8.9)_a_	2,162	(88.9)_a_	54	(2.2)_a_	**< 0.001**
*Foreign*	143	(5.6)	22	(15.2)_b_	112	(78.0)_b_	10	(6.8)_b_	
Years of study									
* < 13 ys*	882	(34.3)	113	(12.8)_a_	742	(84.0)_a_	28	(3.2)_a_	**< 0.001**
*13 ys*	1,262	(49.0)	92	(7.3)_b_	1,148	(91.0)_b_	22	(1.7)_b_	
*>13 ys*	431	(16.7)	33	(7.7)_b_	384	(89.0)_b_	14	(3.3)_a, b_	
Employment status									
*Employed*	985	(38.2)	104	(10.6)_a_	859	(87.2)_a_	22	(2.2)_a_	**0.003**
*Looking for employment*	359	(13.9)	39	(11.0)_a, b_	302	(84.2)_a_	17	(4.9)_b_	
*not in labor force*	1,232	(47.8)	95	(7.7)_b_	1,113	(90.3)_b_	25	(2.0)_a_	
Economic condition at the end of month									
*very easy*	352	(13.7)	64	(18.2)_a_	277	(78.8)_a_	11	(3.1)_a_	**< 0.001**
*quite easy*	1,093	(42.4)	84	(7.7)_b_	989	(90.5)_b_	20	(1.8)_a_	
*some difficulties*	884	(34.3)	64	(7.3)_b_	796	(90.0)_b, c_	24	(2.7)_a_	
*many difficulties*	247	(9.6)	26	(10.7)_b_	213	(85.9)_c_	8	(3.4)_a_	
Geographical area									
*North–West*	518	(20.1)	51	(9.9)	450	(86.7)	18	(3.4)	ns
*North–East*	497	(19.3)	44	(8.8)	446	(89.7)	8	(1.6)	
*Center*	1,040	(40.4)	101	(9.8)	912	(87.7)	26	(2.5)	
*South*	521	(20.2)	42	(8.1)	466	(89.6)	12	(2.3)	
Oral sex									
*Yes*	1,821	(70.7)	205	(11.3)_a_	1,579	(86.7)_a_	37	(2.1)_a_	**< 0.001**
*no/nr*	755	(29.3)	33	(4.4)_b_	695	(92.1)_b_	27	(3.6)_b_	
Anal sex									
*Yes*	991	(38.5)	151	(15.2)_a_	815	(82.2)_a_	25	(2.5)_a_	**< 0.001**
*no/nr*	1,585	(61.5)	87	(5.5)_b_	1,459	(92.1)_b_	39	(2.5)_a_	
Sex in the last year									
No	923	(35.8)	40	(4.3)_a_	857	(92.9)_a_	26	(2.8)_a_	**< 0.001**
Yes	1,653	(64.2)	199	(12.0)_b_	1,417	(85.7)_b_	38	(2.3)_a_	
Condom use									
*Always*	559	(33.8)	55	(9.8)_a_	494	(88.2)_b_	11	(2.0)_a_	**< 0.001**
*Often*	280	(16.9)	38	(13.6)_a_	233	(83.2)_a, c_	9	(3.2)_a_	
Occasionally/sometimes/never	770	(46.6)	100	(13.0)_a_	659	(85.6)_a, b_	11	(1.4)_a_	
*Nr*	44	(2.7)	5	(11.4)_a_	32	(72.7)_c_	7	(15.9)_b_	
Participation in sexuality education meetings									
*No*	1,285	(49.9)	89	(6.9)_a_	1,162	(90.4)_a_	34	(2.6)_a_	**< 0.001**
*Yes*	1,291	(50.1)	149	(11.6)_b_	1,112	(86.1)_b_	30	(2.3)_a_	
*School*	363	(14.1)	50	(13.8)	306	(84.3)	7	(1.9)	
*school and extra school*	44	(1.7)	6	(12.8)	39	(87.2)			
*extra school*	884	(34.3)	94	(10.6)	767	(86.8)	23	(2.6)	
*never*	1,285	(49.9)	89	(6.9)	1,162	(90.4)	34	(2.6)	

Chi-Square Kx3 Test: each letter in subscript format indicates a subset of K covariates whose row proportions are not different at the 0.05 level.

Ns, not significant; nr, not responder.

Condome use: percentages referring to the 1,653 subjects who declared having had sexual intercourse in the last year. Statistically significant values are in bold.

### 3.2 Level of STIs knowledge

Figure 1 shows the 14 STIs-related questions with the absolute and percentage distribution of correct, incorrect, and “I don't know” answers, along with their respective weights. Overall, the average number of weighted correct answers was 5.19 ± 3.17 (37.07% ± 22.64%). Specifically, 27.1% of the sample had a low level of knowledge, 60.4% had a medium level, and 12.5% had a high level (Table 2).

**Table 2:** Level of STIs knowledge.

Level of STIs knowledge	subjects	
	*n*.	(%)
*Low*	699	(27.1)
*Moderate–lower uncertainty recognition*	721	(28.0)
*Moderate–higher uncertainty recognition*	834	(32.4)
*High*	321	(12.5)
***Total***	2,576	**(100.0)**

Statistically significant values are in bold.

**Figure 1:**
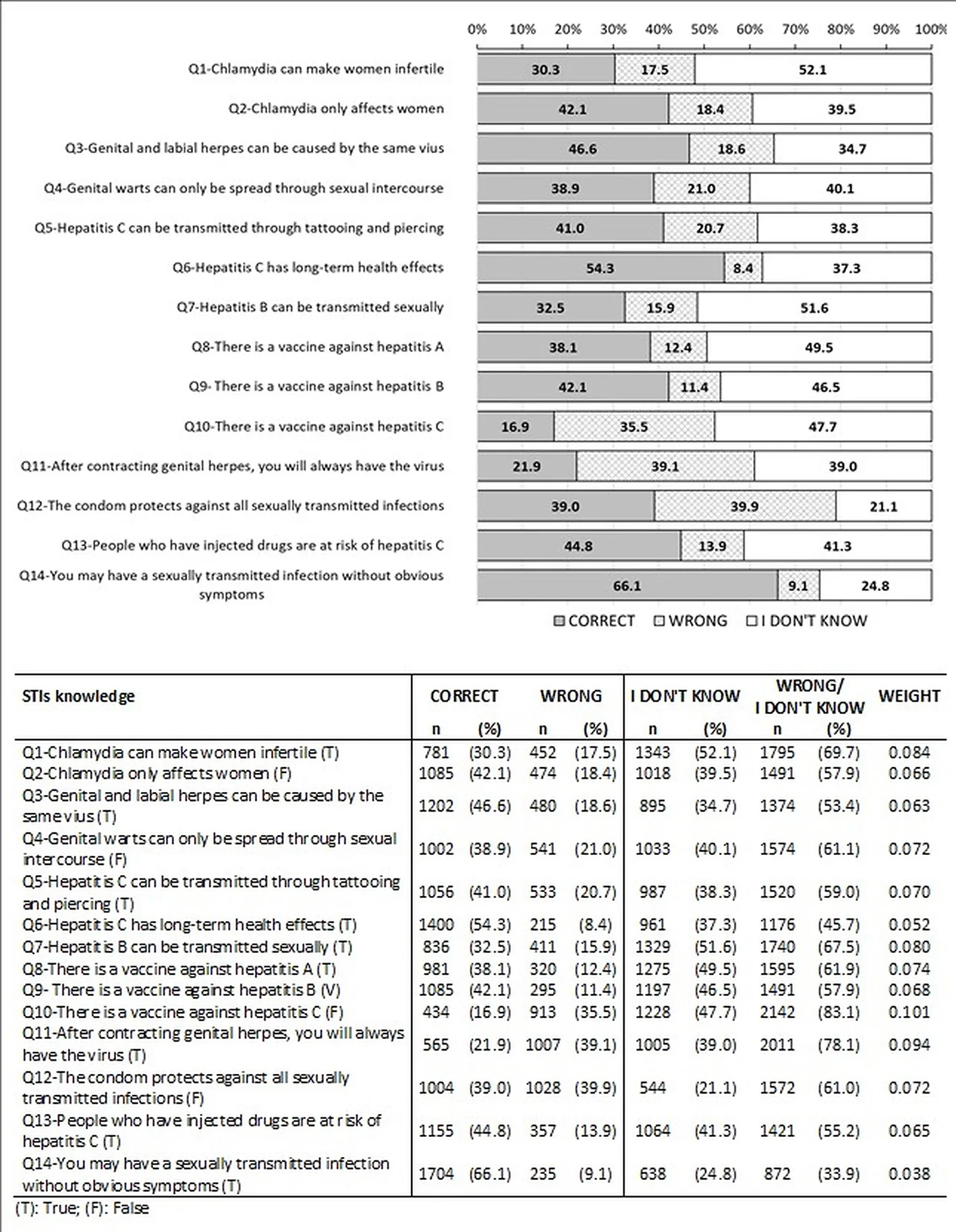
Distribution of STIs knowledge responses.

The questions with the highest difficulty index were Q10 (“There is a vaccine against hepatitis C”) and Q11 (“After contracting genital herpes, you will always have the virus”), with a percentage of incorrect/“I don't know” answers of 83.1% and 78.1%, respectively.

Conversely, the questions with the lowest difficulty index were Q6 (“Hepatitis C has long-term health effects”) and Q14 (“You may have a sexually transmitted infection without obvious symptoms”), with a percentage of correct answers of 54.3% and 66.1%, respectively.

Notably, the questions related to the existence of vaccines against different types of hepatitis (Q8, Q9, Q10) recorded a percentage of “I don't know” answers higher than 40%, while Q12, concerning condoms (“The condom protects against all sexually transmitted infections”), had a percentage of incorrect/“I don't know” answers exceeding 60% (Figure 1).

### 3.3 Factors associated with STIs knowledge

Table 3 shows the average number of weighted correct answers for the main covariates of interest. STIs knowledge was lower among males [35.82% (95%CI: 34.60%−37.04%) vs. 38.41% (95%CI: 37.16%−39.66%)], among younger individuals [29.77% (95%CI: 27.98%−31.57%) vs. 39.15% (95%CI: 38.16%−40.13%)], among job seekers compared to employed individuals [34.02% (95%CI: 31.70%−36.35%) vs. 39.40% (95%CI: 38.00%−40.80%)], among those with significant economic difficulties [32.40% (95%CI: 29.53%−35.27%) vs. 39.17% (95%CI: 36.82%−41.52%)], and among those with lower educational attainment [31.38% (95%CI: 29.93%−32.84%) with < 13 years of education vs. 43.85% (95%CI: 41.74%−45.96%) with >13 years of education].

**Table 3:** STIs weighted correct answers by main covariates—Anova Test.

	STIs weighted corret answers				
	*n*.	(95%CI)	%	(95%CI)	p-value
**Total**	**5.19**	**(5.07–5.31)**	**37.07**	**(36.19–37.95)**	
Sex assigned at birth					
*Males*	5.02	(4.84–5.19)	35.82	(34.60–37.04)	**0.004**
*Females*	5.38	(5.20–5.55)	38.41	(37.16–39.66)	
Gender identity					
*Cisgender*	5.20	(5.08–5.33)	37.15	(36.25–38.04)	ns
*Transgender*	4.93	(4.35–5.51)	35.19	(31.05–39.33)	
Sexual orientation					
*Heterosexual*	5.16	(5.03–5.3)	36.87	(35.9–37.84)	ns
*not Heterosexual*	5.31	(5.03–5.6)	37.96	(35.9–40.02)	
Age group					
*16–19 years*	4.17	(3.92–4.42)	29.77	(27.98–31.57)	**< 0.001**
*20–30 years*	5.48	(5.34–5.62)	39.15	(38.16–40.13)	
Years of study					
* < 13 ys*	4.39	(4.19–4.60)	31.38	(29.93–32.84)	**< 0.001**
*13 ys*	5.42	(5.25–5.60)	38.73	(37.50–39.97)	
*>13 ys*	6.14	(5.84–6.43)	43.85	(41.74–45.96)	
Citizenship					
*Italian*	5.20	(5.07–5.33)	37.14	(36.24–38.04)	ns
*Foreign*	5.03	(4.51–5.56)	35.94	(32.20–39.68)	
Employment status					
*Employed*	5.52	(5.32–5.71)	39.40	(38.00–40.80)	**< 0.001**
*Looking for employment*	4.76	(4.44–5.09)	34.02	(31.70–36.35)	
*not in labor force*	5.05	(4.88–5.23)	36.10	(34.83–37.37)	
Economic condition at the end of month					
*very easy*	5.48	(5.15–5.81)	39.17	(36.82–41.52)	**0.002**
*quite easy*	5.28	(5.09–5.47)	37.71	(36.38–39.04)	
*some difficulties*	5.15	(4.93–5.36)	36.75	(35.25–38.26)	
*many difficulties*	4.54	(4.13–4.94)	32.40	(29.53–35.27)	
Geographical area					
*Nord–West*	5.33	(5.06–5.60)	38.07	(36.12–40.03)	**< 0.001**
*North–East*	5.41	(5.12–5.69)	38.61	(36.60–40.62)	
*Center*	5.33	(5.14–5.52)	38.09	(36.73–39.44)	
*South*	4.56	(4.29–4.83)	32.57	(30.61–34.53)	
Oral sex					
*Yes*	5.60	(5.46–5.74)	40.01	(39.01–41.02)	**< 0.001**
*no/nr*	4.20	(3.97–4.42)	29.97	(28.33–31.60)	
Anal sex					
*Yes*	5.61	(5.41–5.81)	40.07	(38.66–41.49)	**< 0.001**
*no/nr*	4.93	(4.77–5.08)	35.19	(34.09–36.30)	
Condom use in the last year					
*Always*	5.64	(5.38–5.90)	40.26	(38.40–42.12)	**< 0.001**
*Never*	5.92	(5.62–6.23)	42.31	(40.13–44.49)	
*not regular*	5.47	(5.25–5.69)	39.09	(37.52–40.65)	
*no sex*	4.40	(4.19–4.60)	31.40	(29.91–32.89)	
STIs diagnosis					
*Yes*	6.02	(5.65–6.38)	42.99	(40.37–45.60)	**< 0.001**
*No*	5.14	(5.01–5.27)	36.72	(35.79–37.65)	
*Nr*	3.84	(2.97–4.72)	27.46	(21.19–33.72)	
Sexuality education meetings					
*School*	5.57	(5.27–5.88)	39.82	(37.66–41.98)	**< 0.001**
*extra school*	4.76	(4.54–4.98)	34.01	(32.45–35.58)	
*never*	5.36	(5.20–5.53)	38.30	(37.11–39.49)	

Ns, not significant; nr, not responder. Statistically significant values are in bold.

A geographical gradient emerged between the North and the South of the country, with lower knowledge levels in the South [32.57% (95%CI: 30.61%−34.53%)]. Individuals who reported having had an STIs diagnosis and those engaging in oral and anal sex showed higher knowledge levels. Specifically, those who reported an STIs diagnosis answered correctly an average of 42.99% (95%CI: 40.37%−45.60%) of the questions compared to 36.72% (95%CI: 35.79%−37.65%) among those without an STIs diagnosis; those engaging in oral sex answered correctly 40.01% (95%CI: 39.01%−41.02%) vs. 29.97% (95%CI: 28.33%−31.60%), while those engaging in anal sex answered correctly 40.07% (95%CI: 38.66%−41.49%) vs. 35.19% (95%CI: 34.09%−36.30%).

Higher knowledge levels were also observed among those who attended school-based sex education programs compared to those attending extracurricular programs [39.82% (95%CI: 37.66%−41.98%) vs. 34.01% (95%CI: 32.45%−35.58%)] (Table 3, Figure 2).

**Figure 2:**
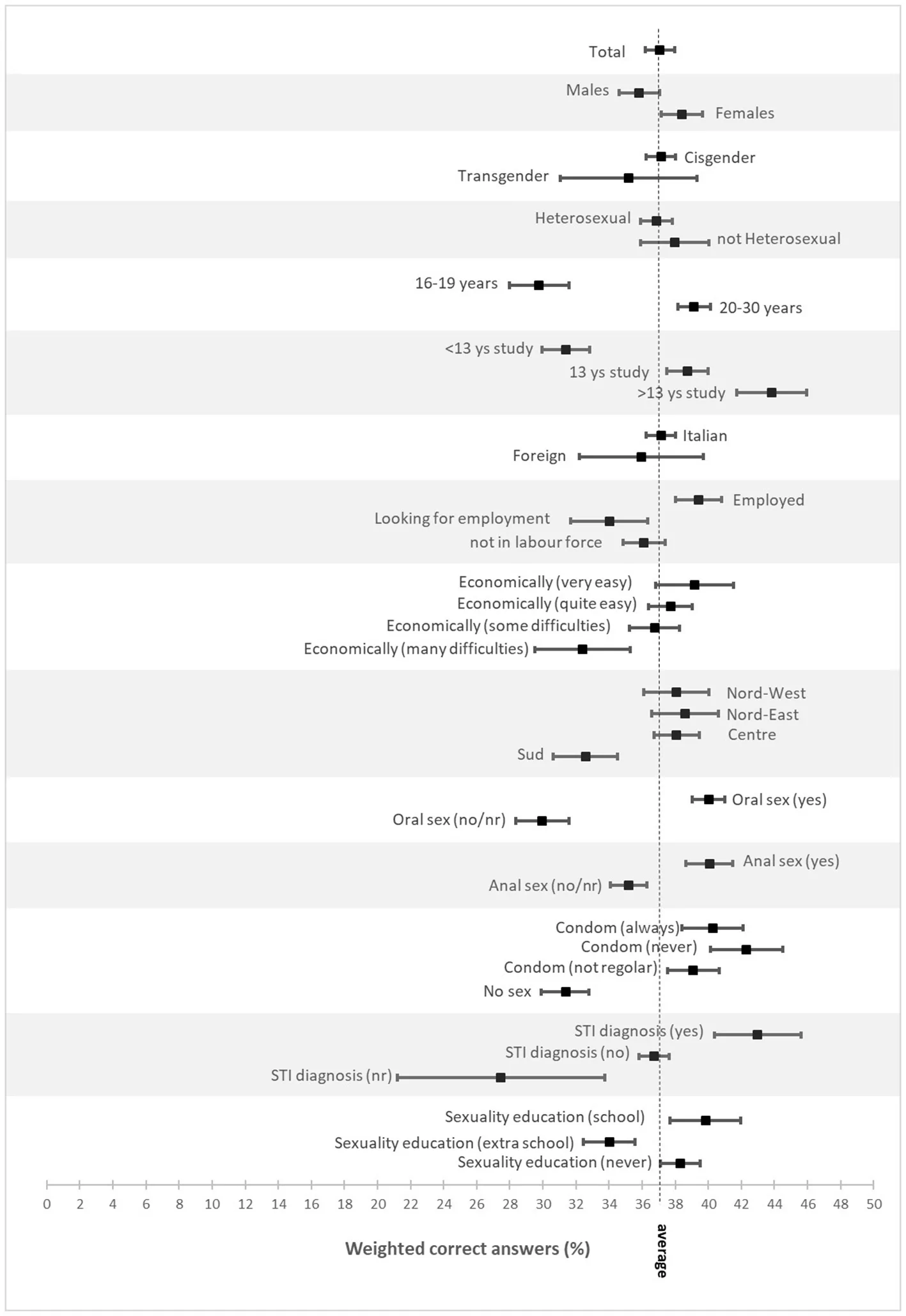
STIs weighted correct answers (%) by main covariates and related 95%CI.

The multivariate analysis, through linear regression shown in Table 4, confirmed that STIs knowledge was lower among males compared to females [β(95%CI):−2.10 (-3.84;−0.37)], among individuals with lower educational attainment compared to those with >13 years of schooling [β(95%CI):−10.13 (-12.91;−7.34] and β(95%CI):−3.42 (-5.9;−0.95)], among those with significant economic difficulties [β(95%CI):−3.88 (-6.82;−0.93)], and in the South of the country compared to the North-East [β(95%CI):−4.39 (-7.15; -1.63)].

**Table 4:** STIs knowledge– Multivariate linear regression.

	STIs knowledge		
	β	(95%CI)	*p*-value
Model constant	35.76	(27.78; 43.74)	0.000
Sex assigned at birth (M vs. F)	**-2.10**	**(**-**3.84;** -**0.37)**	**0.018**
Gender identity (transgender vs. cisgender)	**-**0.80	(-5.54; 3.94)	ns
Sexual orientation (Not hetero vs. hetero)	2.28	(-0.05; 4.6)	ns
Age	**0.51**	**(0.25; 0.78)**	**< 0.001**
< 13ys study (vs. >13ys)	**-10.13**	**(**-**12.91;** -**7.34)**	**< 0.001**
13ys study (vs. >13ys)	**-3.42**	**(**-**5.9;** -**0.95)**	**0.007**
Citizenship (foreign vs. italian)	−2.19	(-6.03; 1.65)	ns
Many economic difficulties (yes vs. no)	-**3.88**	**(**-**6.82;** -**0.93)**	**0.010**
Looking for employment (vs. employed)	−2.04	(-4.83; 0.75)	ns
not in labor force (vs. employed)	−0.37	(-2.52; 1.77)	ns
STIs diagnosis (yes vs. no)	**6.67**	**(3.71; 9.64)**	**< 0.001**
Geographical area (vs. Nord–East)			
Nord–West	−0.12	(-2.84; 2.60)	ns
Center	0.05	(-2.34; 2.43)	ns
South	-**4.39**	**(**-**7.15;** -**1.63)**	**0.002**
Sexuality education (vs. school)			
Never	−2.24	(-4.71; 0.24)	ns
extra school	-**6.51**	**(**-**9.15;** -**3.86)**	**< 0.001**

Y = weighted correct answers (%).

Ns, not significant. Statistically significant values are in bold.

Conversely, knowledge significantly increased with increasing age [β(95%CI): 0.51 (0.25; 0.78)] and was higher among those who reported having received a STIs diagnosis [β(95%CI): 6.67 (3.71; 9.64)].

Regarding participation in sexuality courses, knowledge was lower among those who attended extracurricular courses compared to those who attended school-based courses [β(95%CI):−6.51 (-9.15; -3.86)].

### 3.4 Condom use and reasons for non-use

The question about condom protection against STIs had a percentage of incorrect/“I don't know” answers greater than 60%. Overall, condom use was low; in fact, among the 1,653 (64.2%) young people who reported having had sexual intercourse in the past year, 33.8% (*n* = 559) reported always using condoms, 16.9% (*n* = 280) often, 23.1% (*n* = 382) occasionally/sometimes, and 23.5% (*n* = 388) never. Table 5 shows the percentages of condom use frequency broken down by level of STIs knowledge. The percentage of young people who reported always using condoms was 38.1% among those with a high level of knowledge, 30.5% among those with a medium level without awareness, and 36.2% among those with a low level.

**Table 5:** Condom use by level of STIs knowledge.

	Total (*n*.2,576)		Low level (*n*.699)		Moderate-lower uncertainty recognition (*n*.721)		Moderate-higher uncertainty recognition (*n*.834)		High (*n*.321)	
	*n*.	(%)	*n*.	(%)	*n*.	(%)	*n*.	(%)	*n*.	(%)
Sex in the last year										
No	923	(35.8)	343	(49.1)	225	(31.2)	272	(32.6)	83	(25.9)
Yes	1,653	(64.2)	356	(50.9)	496	(68.8)	563	(67.4)	238	(74.1)
Condom use										
Always	559	(33.8)	129	(36.2)	151	(30.5)	188	(33.5)	91	(38.1)
Often	280	(16.9)	53	(14.8)	102	(20.5)	87	(15.5)	37	(15.8)
Occasionally/ sometimes/ never	770	(46.6)	154	(43.2)	232	(46.9)	277	(49.3)	106	(44.6)
nr	44	(2.7)	20	(5.7)	10	(2.0)	10	(1.7)	4	(1.5)

Nr, not responder.

Figures 3a, 3b illustrates the reasons for not using condoms among the 770 subjects who reported never or only occasionally/sometimes using them in the past year.

**Figure 3:**
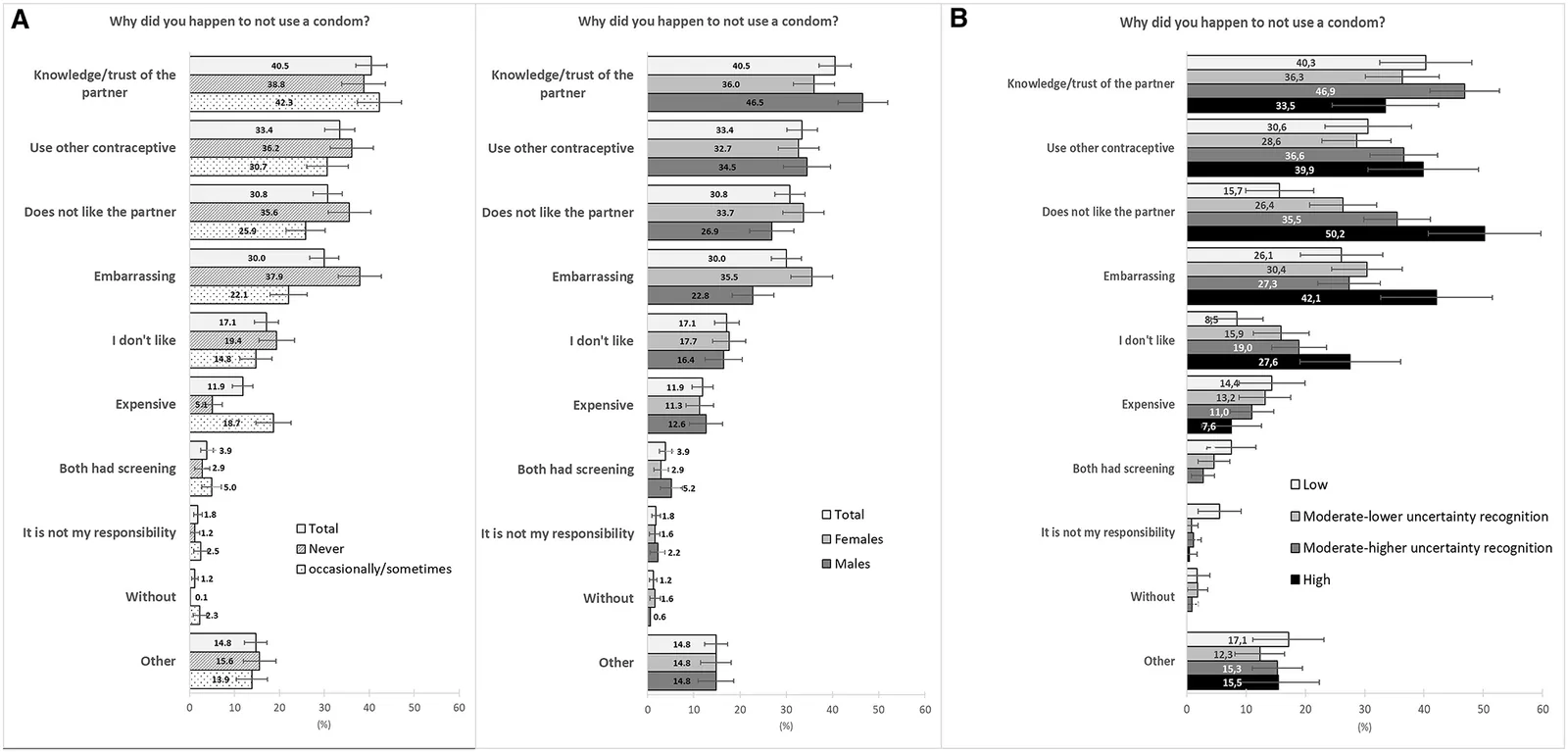
**(a)** Reasons for not using condoms, by frequency of use and sex assigned to birth. **(b)** Reasons for not using condoms, by level of STIs knowledge.

A total of 40.5% (*n* = 312) did not use condoms because they trusted and knew their partner, 30.8% (*n* = 237) because their partner disliked them, 33.4% (*n* = 257) because they used another contraceptive method, and 30.0% (*n* = 231) because they considered it embarrassing. Moreover, for 11.9% (*n* = 91), condoms were considered expensive, with a higher percentage among those who used them occasionally/sometimes [18.7% (95%CI: 14.8%−22.6%) vs. 5.1% (95%CI: 2.9%−7.3%)]. Conversely, the frequency of non-use due to embarrassment or because their partner disliked them was higher among those who never used condoms compared to those who used them occasionally/sometimes [37.9% (95%CI: 33.1%−42.7%) vs. 22.1% (95%CI: 17.9%−26.2%) and 35.6% (95%CI: 30.9%−40.4%) vs. 25.9% (95%CI: 21.5%−30.3%), respectively]. For 1.8% (*n* = 14) of respondents, using condoms was not considered their responsibility (Figure 3a).

When disaggregating by sex assigned at birth, non-use of condoms due to trust and knowledge of the partner was more frequent among males [46.5% (95%CI: 41.1%−51.9%) vs. 36.0% (95%CI: 31.5%−40.5%)], whereas non-use due to embarrassment was more frequent among females [35.5% (95%CI: 31.0%−39.9%) vs. 22.8% (95%CI: 18.3%−27.3%)] (Figure 3a). Furthermore, regarding sex at birth, females were more likely than males not to use condoms because their partner disliked them (33.7% vs. 26.9%), although this difference was not significant (Figure 3a).

Regarding STIs knowledge levels, young people with high levels of knowledge mainly reported not using condoms because their partner disliked them (50.2%), due to embarrassment (42.1%), and because they themselves disliked them (27.6%), with significantly higher differences compared to those with low or medium levels of knowledge. Specifically, the frequency of non-use because the partner disliked condoms was 50.2% (95%CI: 40.7%−59.7%) among those with high knowledge, 26.4% (95%CI: 20.7%−32.0%) among those with medium knowledge without awareness, and 15.7% (95%CI: 9.9%−21.4%) among those with low knowledge. Non-use due to embarrassment was reported by 42.1% (95%CI: 32.7%−51.5%) of those with high knowledge vs. 27.3% (95%CI: 22.1%−32.6%) of those with medium knowledge with awareness; non-use because they themselves disliked condoms was reported by 27.6% (95%CI: 19.1%−36.1%) of those with high knowledge vs. 8.5% (95%CI: 4.1%−12.9%) of those with low knowledge. Non-use due to cost was more frequent among those with low knowledge (14.4% and 5.6%, respectively), without significant differences between knowledge levels (Figure 3b).

### 3.5 Factors associated with occasional or no condom use

The multivariate analysis compared participants who reported always using condoms (*n* = 559) with those who reported using them occasionally or sometimes, or never (*n* = 770). Occasional or no condom use was significantly higher among females [adjOR (95%CI): 1.75 (1.37–2.23)], among those with lower education levels [adjOR (95%CI): 2.00 (1.37–2.92) and adjOR (95%CI): 1.73 (1.27–2.38)], among employed individuals [adjOR (95%CI): 1.77 (1.22–2.55)] compared to job seekers, among those with economic difficulties [adjOR (95%CI): 1.75 (1.37–2.24)], and among those engaging in oral sex [adjOR (95%CI): 2.42 (1.24–4.72)] and anal sex [adjOR (95%CI): 2.28 (1.78–2.93)]. No significant correlation emerged between condom use and STIs knowledge level (Table 6).

**Table 6:** Condom use among those who reported having sex in the last year—Logistic regression.

	Total	not use of condoms (occasionally/sometimes/never VS always)			
	*n*.	*n*.	(%)	adjOR (95%CI)	p
**Total**	1,329^*^	**770**	**(100.0)**		
Sex assigned at birth					
*Males*	641	331	(51.7)	ref	
*Females*	689	439	(63.7)	**1.75 (1.37–2.23)**	**< 0.001**
Gender identity					
*Cisgender*	1,272	739	(58.1)	ref	
*Transgender*	58	31	(53.6)	0.75 (0.42–1.35)	ns
Sexual orientation					
*Heterosexual*	1,129	650	(57.6)	ref	
*not Heterosexual*	201	120	(59.7)	1.01 (0.72–1.44)	ns
**Age**	24.0 ± 3.8	24.2 ± 3.8		1.03 (0.99–1.06)	ns
Citizenship					
*Italian*	1,267	731	(57.7)	ref	
*Foreign*	62	38	(62.2)	1.04 (0.58–1.87)	ns
Years of study					
* < 13ys*	380	239	(62.9)	**2.00 (1.37–2.92)**	**< 0.001**
*13ys*	687	404	(58.8)	**1.73 (1.27–2.38)**	**0.001**
*>13ys*	262	127	(48.5)	ref	
Employment status					
*Employed*	630	387	(61.4)	**1.77 (1.22–2.55)**	**0.003**
*Looking for employment*	192	100	(52.1)	ref	
*not in labor force*	508	283	(55.8)	1.45 (1.00–2.11)	ns
Economic difficulties^∧^					
Yes	601	396	(65.9)	**1.75 (1.37–2.24)**	**< 0.001**
No	729	374	(51.4)	ref	
Oral sex					
*Yes*	1,221	728	(59.6)	**2.42 (1.24–4.72)**	**0.010**
*No*	47	14	(30.1)	ref	
*Nr*	61	28	(45.0)	1.14 (0.42–3.07)	ns
Anal sex					
*Yes*	712	475	(66.8)	**2.28 (1.78–2.93)**	**< 0.001**
*No*	558	260	(46.6)	ref	
*Nr*	59	34	(57.6)	**2.67 (1.23–5.82)**	**0.013**
STIs diagnosis					
*Yes*	155	100	(64.7)	1.15 (0.78–1.69)	ns
*No*	1,153	659	(57.2)	ref	
*Nr*	22	11	(49.1)	0.7 (0.28–1.75)	ns
Level of STIs knowledge					
*Low*	283	154	(54.4)	1.09 (0.74–1.62)	ns
*Moderate*–*lower uncertainty recognition*	384	232	(60.5)	1.41 (0.97–2.04)	ns
*Moderate*–*higher uncertainty recognition*	466	277	(59.6)	1.40 (0.98–2.00)	ns
*High*	197	106	(54.0)	ref	
Geographical area					
*Nord*–*West*	297	189	(63.6)	**1.68 (1.16–2.44)**	**0.006**
*North*–*East*	239	139	(58.4)	1.21 (0.82–1.78)	ns
*Center*	546	309	(56.5)	1.20 (0.87–1.66)	ns
*South*	248	133	(53.7)	ref	

*subjects who often used condoms were excluded.

^∧^Economic difficulties (yes: some and many difficulties at the end of month; no: condition at the end of month very and quite easy).

Nr, not responder; ns, not significant; ref, reference. Statistically significant values are in bold.

## 4 Discussion

Consistent with previous surveys, overall STIs knowledge in our sample appears modest; 12.5% of participants attained a high score. Infact, a study conducted between 2013 and 2014 on 2,880 students from 21 different secondary schools in Italy revealed a significant cultural gap in terms of sexual health and prevention (18). Another european study conducted in 2020 among young individuals concerning STIs, showed an insufficient level of knowledge (estimated knowledge at 45.97%)—still higher than ours (12%) (25).

Knowledge about condoms as a preventive measure is also inadequate, with over 60% of respondents answering incorrectly or “I don't know” to the question about protection against STIs. These results are alarming, especially when compared with the global and national increase in STIs incidence—such as chlamydia, gonorrhea, and syphilis—reported by the WHO and other recent epidemiological studies (11, 12, 26).

Indeed, the low awareness of STIs among young people could suggest an increase in these infections, as also highlighted by the Italian National Institute of Health (12); however, due to the cross-sectional design of the present study, it is not possible to draw conclusions on a possible relationship between knowledge of STIs and the trend of their incidence.

In this paper, the multivariable analysis showed that gender identity, age, educational level, and economic conditions were significantly associated with STIs knowledge, with lower scores among males, younger respondents, those with fewer years of education, and those reporting economic hardship. For example, women demonstrated greater knowledge than men—this discrepancy could likely be linked to their more direct exposure to reproductive health campaigns. Age appears to be a determining factor, in effect knowledge increases with age, probably due to greater life experience or access to more information sources. A knowledge gap between Northern and Southern Italy also emerged, with lower levels in the South. This geographical inequality clearly requires specific and targeted interventions in certain Regions.

Beyond the knowledge gap, the study shows that only 33.8% of sexually active youth in the past year reported always using condoms, while 46.6% used them rarely or never. The main reasons for non-use were trust in their partner (40.5%), discomfort or embarrassment (30%), and the fact that the partner disliked it (30.8%). However, even among young people with high knowledge levels, not using condoms was often linked to emotional or relationship-related reasons—such as the partner's dislike (50.2%) or embarrassment (42.1%). Similar studies have shown low condom use among young adults, with only one-third of the sample consistently using them (22).

In our sample, STIs knowledge level was not significantly associated with occasional or no condom use. Participants nevertheless reported several psychological, cultural, and relational reasons for not using condoms. Given the cross-sectional design of the study, these findings do not allow us to determine whether changes in STIs knowledge would lead to changes in condom-use behavior.

The psychological and relational dimensions of sexual health are also relevant when considering the broader consequences of STIs. Previous studies have indicated that STIs testing and diagnosis may be associated with stigma, anxiety, depressive symptoms, relationship difficulties, and adverse effects on quality of life and sexual functioning. In particular, Ciccarese et al. found more frequent and severe depressive symptoms and slightly poorer sexual functioning among patients with STIs other than HIV than among patients with chronic inflammatory bowel diseases. Other evidence has similarly highlighted depressive symptoms and quality-of-life concerns among people with chronic STIs, as well as fear, embarrassment, isolation, and relationship strain associated with STIs testing and diagnosis (14–16). Although these dimensions were not directly assessed in the present study, they should be considered in future research and sexual-health initiatives.

Approximately half of the participants reported having attended sexuality education programs. Participants who attended school-based programs had higher STIs knowledge scores than those who attended programs delivered in non-school settings. However, because of the cross-sectional design, this association should not be interpreted as evidence that school-based sexuality education caused the observed difference in knowledge. Unmeasured individual, educational, or contextual factors may also have contributed to this finding. School-based sexuality education may nevertheless represent a potential strategy for improving STIs knowledge, but its effectiveness should be assessed through longitudinal or intervention studies.

Another critical aspect involves economic barriers: 11.9% of young people who reported not using condoms cited cost as a reason for not using them. Therefore, providing free contraceptives should be considered as a strategy to remove this barrier (27, 28).

To improve STIs knowledge and modify risky behaviors, several strategic actions are warranted. A coordinated intervention involving schools, health authorities, and community organizations is essential. Mandatory sexual education in all secondary schools—with updated, multidisciplinary content that addresses gender differences and regional disparities—and the involvement of specialists would be beneficial. Specialists can provide technical input to educators, who then translate content into effective pedagogies. Training educators (and, where feasible, the general public) to recognize and counter potential sources of misinformation about STIs would further strengthen these efforts (29).

It is worth emphasizing that interpreting our findings requires caution against equating factual knowledge with protective behavior. Effective interventions should strengthen not only access to information but also critical appraisal and the skills needed to navigate digital and interpersonal contexts. In particular, eHealth/media literacy—defined as the ability to find, evaluate, and apply online health information—is pivotal in environments saturated with conflicting or misleading sexual-health content. Consequently, school-, community-, and media-based programs that cultivate critical competencies and supportive contexts are more consistent with contemporary health-literacy theory than approaches focused solely on correcting individual deficits. Building on this perspective, addressing geographical disparities in STIs knowledge through targeted interventions in disadvantaged regions—with dedicated funding for training educational and healthcare staff—would help bridge gaps. Informational campaigns that tackle psychological barriers to condom use (e.g., embarrassment, low assertiveness in relationships) can further reinforce protective behaviors.

These findings support the need for comprehensive sexual health strategies that go beyond the simple transmission of knowledge. Recent evidence from Italy has shown that distinct STI/HPV information-source profiles among young adults are associated with different preventive orientations, reinforcing the need for integrated and targeted sexual health communication strategies (30).

### 4.1 Limitations and strengths

This study has some limitations. First, because of its cross-sectional and observational design, the temporal direction of the observed associations could not be established, and no causal inferences can be drawn regarding the relationships between STIs knowledge, participation in sexuality education programs, and condom-use behavior. Second, the questionnaire was based on self-reported responses, which carry the risk of underreporting or overreporting behaviors and knowledge. Discussing sexual activity, intimate life, and conditions still subject to social stigma—even anonymously—may have led participants to approximate their answers. Furthermore, the geographical distribution of the participants may not be representative of the entire Italian population, highlighting the need for further studies with larger and more diverse samples. The exclusion of non-binary individuals also may limit the generalizability of the findings to this category.

Nevertheless, this study also presents several strengths. First, it involved a large and geographically diverse sample of young people from various regions of Italy, allowing for the identification of significant socio-demographic patterns, such as regional disparities. Second, the questionnaire was specifically designed not only to assess STIs knowledge, but also to explore behavioral and emotional-relational factors influencing condom use, thus providing a comprehensive picture of young people's attitudes toward sexual health. Finally, the study addressed emerging and highly relevant issues—such as emotional barriers to condom use—offering valuable insights for the development of more effective public health strategies.

The findings highlight gaps in STIs knowledge among the young people included in the study. Although the present results do not establish the effectiveness of specific educational interventions, schools may represent an important setting in which comprehensive sexual and emotional education programs could be developed and evaluated, alongside healthcare and community settings.

It is essential to raise awareness and provide information not only from a medical-scientific perspective but also from a psychological one, to increase STIs-related knowledge and risk awareness, promote a culture of mutual respect and gender equality, and spread the understanding that psychological motivations should not override individual or partner health risks. To achieve this, multidisciplinary interventions involving teachers, healthcare professionals, and psychologists should be organized and implemented to equip young people with the necessary knowledge for individual and collective prevention.

## Data Availability

The data are available from the corresponding author upon reasonable request.
